# Supplementary material for: Microbial Hotspots in Lithic Microhabitats Inferred from DNA Fractionation and Metagenomics in the Atacama Desert
Source: Microorganisms. 2021 May 12;9(5):1038. doi: 10.3390/microorganisms9051038 (PMC8151210; doi:10.3390/microorganisms9051038)
Supplement: Supplementary file 1 [file microorganisms-09-01038-s001.zip › microorganisms-1184168-supplementary.pdf]

Supplemental Material

Figures S1–S4:

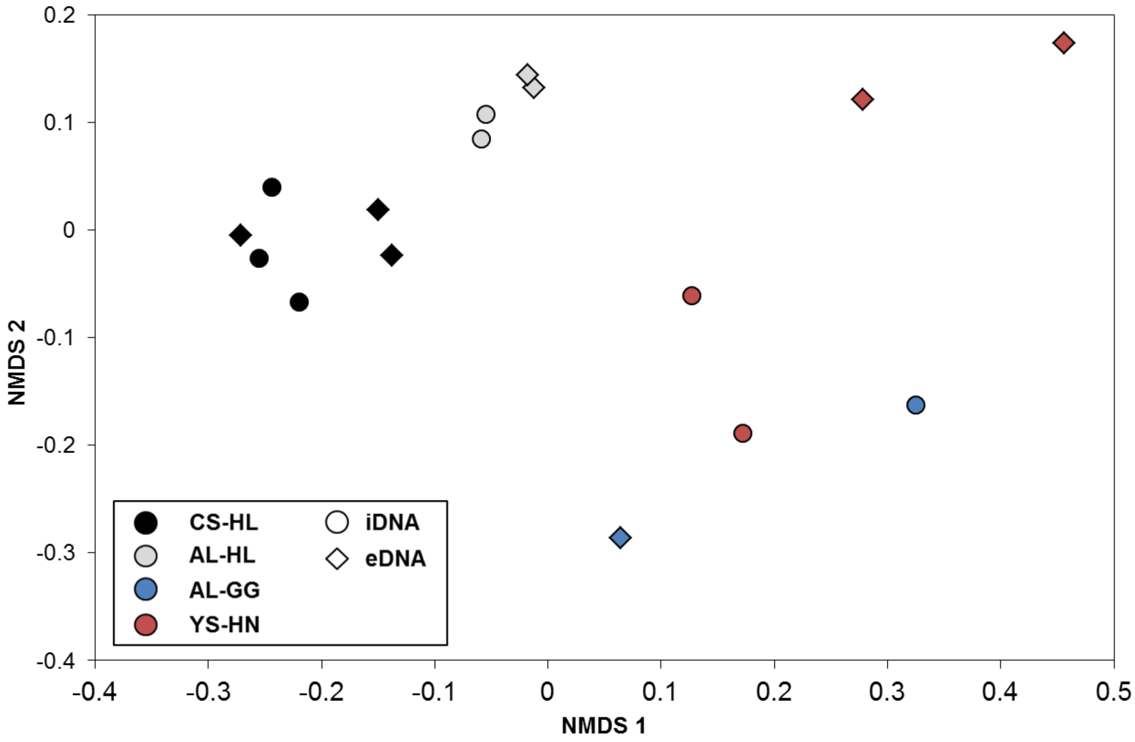

Figure S1: Nonmetric multidimensional scaling plot showing differences between of microbial eDNA and iDNA communities from four different Atacama Desert location representing three habitat types. CS-HL and Al-HL samples represent hypolith communities, AL-GG depicted gypsum crusts community and YS/HN depicts halite nodules communities

Phylum	Genus	CS-HL		AL-HL		AL-GG		YS-HN	
		eDNA	iDNA	eDNA	iDNA	eDNA	iDNA	eDNA	iDNA
Euryarchaeota	Halococcus	0.000	0.000	0.000	0.000	0.002	0.000	0.015	0.244
	Halomarina	0.000	0.000	0.000	0.000	0.000	0.000	0.000	0.023
	Natronomonas	0.000	0.000	0.000	0.000	0.000	0.000	0.000	0.011
	Unclassified Halomicrobiaceae	0.000	0.000	0.000	0.000	0.000	0.000	0.000	0.046
	Unclassified Halobacteriales	0.000	0.000	0.000	0.000	0.002	0.000	0.000	0.103
Thaumarchaeota	Unclassiffied Nitrososphaeraceae	0.000	0.000	0.000	0.007	0.000	0.013	0.000	0.000
Actinobacteria	Unclassified Actinomarinales	0.000	0.000	0.000	0.000	0.000	0.000	0.000	0.009
	Uncultured Acidimicrobiia IMCC26256	0.002	0.006	0.001	0.001	0.000	0.001	0.000	0.001
	Acidimicrobiia IMCC26256	0.002	0.001	0.000	0.027	0.000	0.055	0.000	0.000
	Uncultured Acidimicrobiia	0.014	0.013	0.003	0.017	0.000	0.033	0.001	0.000
	Nocardia	0.004	0.013	0.039	0.013	0.007	0.009	0.026	0.004
	Jatrophihabitans	0.002	0.001	0.015	0.004	0.000	0.000	0.000	0.000
	Blastococcus	0.006	0.003	0.004	0.032	0.000	0.063	0.000	0.000
	Uncultured Frankiales	0.007	0.004	0.004	0.027	0.000	0.053	0.002	0.000
	Quadrisphaera	0.010	0.003	0.001	0.000	0.000	0.000	0.000	0.000
	Brachybacterium	0.000	0.000	0.000	0.001	0.038	0.001	0.001	0.000
	Unclassified Intrasporangiaceae	0.000	0.000	0.000	0.000	0.002	0.000	0.014	0.000
	Microbacterium	0.000	0.000	0.000	0.000	0.011	0.000	0.009	0.000
	Kribbella	0.007	0.002	0.000	0.000	0.000	0.000	0.000	0.000
	Nocardioides	0.015	0.008	0.009	0.002	0.001	0.000	0.002	0.000
	Cutibacterium	0.001	0.000	0.001	0.000	0.011	0.001	0.012	0.003
	Unclassified Propionibacteriaceae	0.000	0.000	0.006	0.000	0.000	0.000	0.000	0.000
	Crossiella	0.001	0.000	0.000	0.015	0.000	0.029	0.000	0.000
	Uncultured Euzebyaceae	0.009	0.004	0.012	0.004	0.000	0.000	0.000	0.001
	Rubrobacter	0.100	0.049	0.017	0.019	0.000	0.027	0.004	0.001
	Conexibacter	0.023	0.022	0.002	0.019	0.000	0.038	0.001	0.000
	Solirubrobacter	0.011	0.004	0.101	0.024	0.000	0.021	0.001	0.000
	Unclassified Solirubrobacteraceae	0.004	0.001	0.039	0.011	0.000	0.019	0.000	0.000
Bacteroidetes	Segetibacter	0.002	0.003	0.004	0.070	0.000	0.133	0.000	0.000
	Tunicatimonas	0.011	0.007	0.000	0.000	0.000	0.000	0.000	0.000
	Uncultured Cyclobacteriaceae	0.009	0.003	0.000	0.000	0.000	0.000	0.000	0.000
	Rhodocytophaga	0.018	0.010	0.002	0.002	0.000	0.000	0.000	0.000
	Adhaeribacter	0.008	0.005	0.002	0.001	0.000	0.000	0.000	0.000
	Hymenobacter	0.000	0.000	0.007	0.000	0.000	0.000	0.000	0.000
	Pontibacter	0.007	0.010	0.000	0.000	0.000	0.000	0.000	0.000
	Unclassified Cytophagales	0.000	0.000	0.012	0.002	0.000	0.000	0.000	0.000
	Chryseobacterium	0.000	0.000	0.000	0.000	0.034	0.000	0.001	0.000
	Cloacibacterium	0.001	0.000	0.000	0.001	0.037	0.001	0.031	0.001
	Pedobacter	0.007	0.003	0.000	0.000	0.000	0.000	0.000	0.000
	Salinibacter	0.000	0.000	0.000	0.000	0.004	0.000	0.000	0.131
Chloroflexi	Uncultured Kallotenuales AKIW781	0.009	0.003	0.024	0.082	0.000	0.099	0.000	0.000
	Thermobaculum	0.004	0.003	0.002	0.014	0.000	0.028	0.000	0.000
	Uncultured Thermomicrobiales AKYG1722	0.013	0.004	0.000	0.000	0.000	0.000	0.000	0.000
	Uncultured Thermomicrobiales JG30-KF-CM45	0.064	0.018	0.038	0.020	0.000	0.022	0.001	0.000
Cyanobacteria	Aliterella CENA595	0.290	0.526	0.026	0.017	0.000	0.002	0.004	0.000
	Gloeocapsa PCC-7428	0.023	0.017	0.000	0.000	0.000	0.000	0.000	0.000
	Uncultured Chroococcidiopsaceae	0.006	0.006	0.000	0.000	0.000	0.000	0.000	0.000
	Unclassified Chroococcidiopsaceae	0.016	0.019	0.430	0.233	0.000	0.000	0.003	0.001
	Uncultured Nostocales	0.005	0.010	0.008	0.052	0.000	0.000	0.000	0.000
	Uncultured Nostocales	0.000	0.000	0.007	0.040	0.000	0.000	0.000	0.000
	Unclassified Nostocales	0.009	0.005	0.001	0.000	0.005	0.000	0.000	0.316
	Unclassified Oxyphotobacteria	0.044	0.043	0.021	0.002	0.000	0.000	0.001	0.004
Deinococcus-Thermus	Deinococcus	0.000	0.000	0.000	0.001	0.000	0.000	0.017	0.000
	Truepera	0.038	0.039	0.014	0.005	0.000	0.001	0.001	0.000
Firmicutes	Anoxybacillus	0.001	0.000	0.000	0.001	0.001	0.001	0.062	0.000
	Unclassified Ruminococcaceae	0.000	0.000	0.000	0.000	0.000	0.000	0.009	0.000
Gemmatimonadetes	Uncultured Gemmatimonadaceae	0.000	0.000	0.000	0.000	0.000	0.000	0.008	0.000
Patescibacteria	Uncultured Saccharimonadales	0.005	0.002	0.000	0.006	0.000	0.013	0.000	0.000
Proteobacteria	Roseomonas	0.000	0.001	0.001	0.001	0.000	0.001	0.011	0.000
	Caulobacter	0.000	0.000	0.000	0.001	0.065	0.002	0.004	0.000
	Caulobacteraceae PMMR1	0.012	0.010	0.000	0.000	0.000	0.000	0.000	0.000
	Unclassified Caulobacteraceae	0.002	0.002	0.000	0.000	0.000	0.000	0.038	0.000
	Methylobacterium	0.001	0.002	0.002	0.020	0.138	0.038	0.009	0.003
	Rhizobium	0.003	0.002	0.000	0.000	0.025	0.000	0.005	0.000
	Unclassified Xanthobacteraceae	0.001	0.001	0.002	0.004	0.031	0.006	0.002	0.001
	Uncultured Rhizobiales	0.019	0.009	0.004	0.001	0.000	0.000	0.000	0.000
	Paracoccus	0.000	0.000	0.001	0.006	0.001	0.011	0.028	0.002
	Sphingomonas	0.005	0.003	0.005	0.006	0.003	0.010	0.001	0.001
	Uncultured Sphingomonadaceae	0.008	0.008	0.001	0.000	0.002	0.000	0.000	0.000
	Unclassified Sphingomonadaceae	0.027	0.022	0.001	0.011	0.000	0.023	0.001	0.000
	Acidovorax	0.000	0.000	0.000	0.000	0.035	0.000	0.018	0.002
	Delftia	0.000	0.000	0.000	0.007	0.000	0.014	0.000	0.000
	Herbaspirillum	0.000	0.000	0.000	0.000	0.041	0.000	0.000	0.000
	Methylobacillus	0.000	0.000	0.000	0.000	0.014	0.000	0.000	0.000
	Unclassified Methylophilaceae	0.005	0.009	0.013	0.081	0.300	0.150	0.302	0.015
	Uncultured Neisseriaceae	0.000	0.000	0.000	0.000	0.001	0.000	0.000	0.010
	Enterobacter	0.000	0.000	0.000	0.000	0.000	0.000	0.006	0.000
	Acidibacter	0.000	0.000	0.000	0.000	0.000	0.000	0.005	0.000
	Acinetobacter	0.002	0.002	0.003	0.005	0.064	0.008	0.160	0.002
	Enhydrobacter	0.000	0.001	0.001	0.000	0.001	0.000	0.010	0.001
	Pseudomonas	0.001	0.001	0.001	0.003	0.002	0.006	0.099	0.004
	Uncultured Steroidobacteraceae	0.000	0.000	0.000	0.000	0.015	0.000	0.000	0.000
	Pseudoxanthomonas	0.000	0.000	0.000	0.000	0.050	0.000	0.000	0.000
Verrucomicrobia	Candidatus Udaeobacter	0.006	0.001	0.000	0.000	0.000	0.000	0.000	0.000
Unassigned	Unassigned	0.001	0.002	0.000	0.001	0.000	0.000	0.021	0.000

Figure S2: Genus level taxonomy depicting relative abundances of the eDNA and iDNA pools across the four sampled habitats

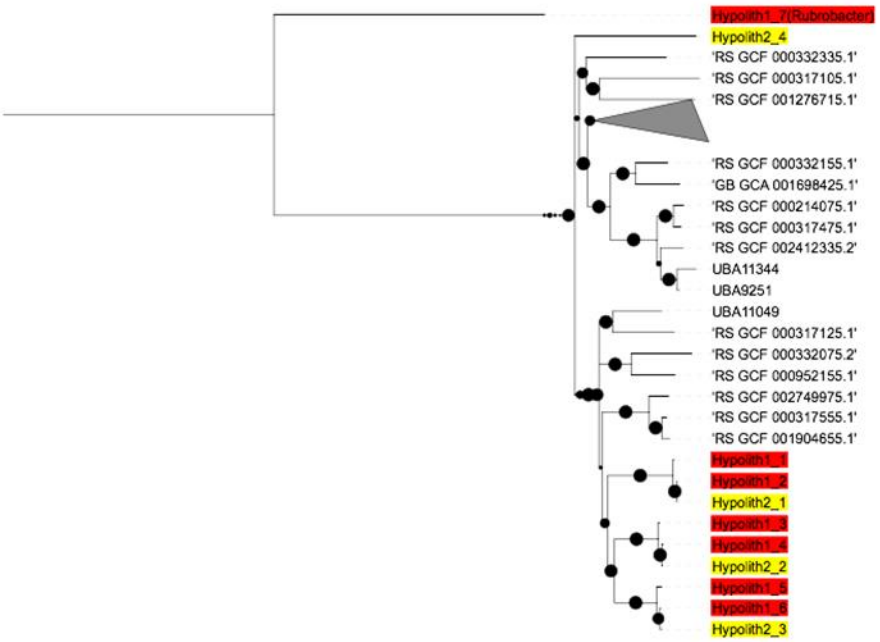

Figure S3: Phylogenetic tree showing classification of 16S rRNA genes recovered from metagenome assembled genomes (MAGs) reconstructed from hypolith samples

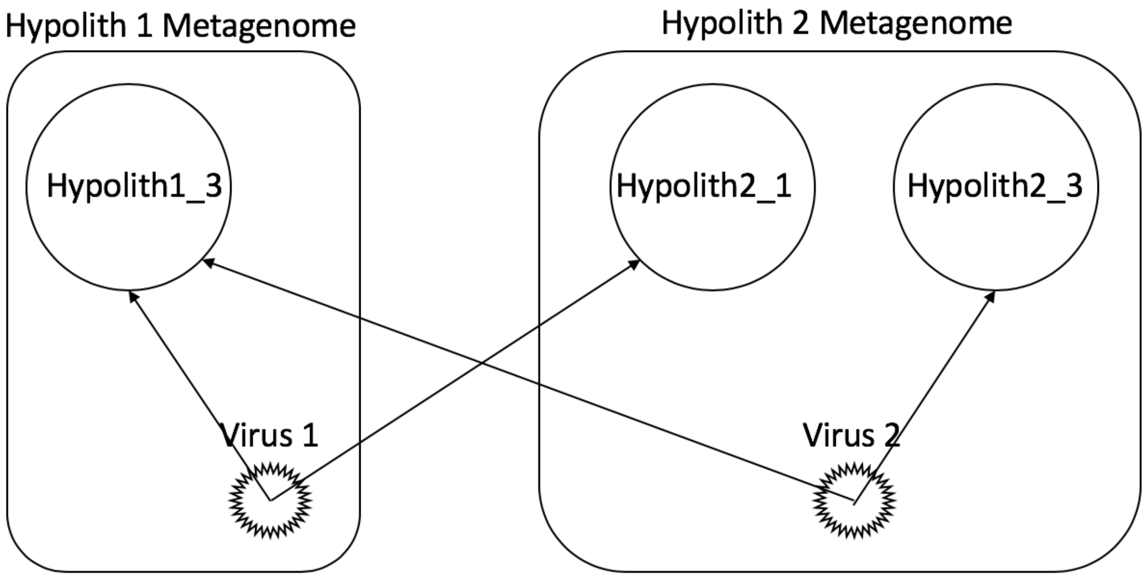

Figure S4: Visualization of putative host-virus interaction across two hypolith metagenomes. An arrow between a virus and a genome indicates that a spacer to viral contig match was found

Tables S1–S3:

Table S1: Sequence and alpha diversity measurements

Sample	Generated sequences	Sequences post trimming and contamination removal	Number of ASVs (rarefied to 69,772 seqs)	Shannon Diversity Index (rarefied to 69,772 seqs)
CS-HL eDNA Rep 1	521,452	188,376	729	4.47
CS-HL eDNA Rep 2	494,292	264,312	667	4.58
CS-HL eDNA Rep 3	510,660	291,928	558	3.76
CS-HL iDNA Rep 1	325,916	168,192	587	2.85
CS-HL iDNA Rep 2	793,588	503,980	642	4.01
CS-HL iDNA Rep 3	617,700	391,548	568	2.40
AL-HL eDNA Rep 1	819,564	598,292	525	3.57
AL-HL eDNA Rep 2	697,448	411,884	565	3.45
AL-HL iDNA Rep 1	626,244	421,228	506	3.26
AL-HL iDNA Rep 2	778,264	456,408	392	2.51
AL-GG eDNA	263,188	69,772	207	3.02
Al-GG iDNA	241,720	107,788	297	3.45
YS-HN eDNA Rep 1	326,976	151,660	289	3.39
YS-HN eDNA Rep 2	395,972	140,512	358	2.43
YS-HN iDNA Rep 1	560,532	282,920	378	2.91
YS-HN iDNA Rep 2	280,636	280,636	199	2.69

Table S2. Estimation of Metagenome Assembled Genome (MAG) quality and RpS3 protein based taxonomy for all MAGs studied

	CheckM Statistics			Taxonomy (based on Ribosomal Protein S3)
MAG	Completeness (%)	Contamination (%)	Strain heterogeneity (%)	
Hypolith1_1	99.26	2.67	7.69	Bacteria;Cyanobacteria;Chroococcidiopsidales;Chroococcidiopsidaceae;Chroococcidiopsis
Hypolith1_2	93.48	3.37	13.04	Bacteria;Cyanobacteria;unclassified;unclassified;unclassified;
Hypolith1_3	82.11	2.30	23.08	Bacteria;Cyanobacteria;Chroococcidiopsidales;Chroococcidiopsidaceae;Chroococcidiopsis
Hypolith1_4	82.97	0.79	0.00	Bacteria;Actinobacteria;Rubrobacteria;Rubrobacterales;Rubrobacter;
Hypolith1_5	69.46	1.66	0.00	Bacteria;Cyanobacteria;Oscillatoriophycideae;Oscillatoriales;unclassified;
Hypolith1_6	66.54	0.44	0.00	Bacteria;Actinobacteria;Rubrobacteria;Rubrobacterales;Rubrobacter;
Hypolith1_7	49.53	2.36	0.00	Bacteria;Chloroflexi;unclassified;unclassified;unclassified;
Hypolith1_8	33.45	0.00	0.00	No rpS3
Hypolith2_1	99.41	3.84	9.09	Bacteria;Cyanobacteria;Chroococcidiopsidales;Chroococcidiopsidaceae;Chroococcidiopsis
Hypolith2_2	90.74	0.71	0.00	Bacteria;Cyanobacteria;Oscillatoriophycideae;Oscillatoriales;Oscillatoriaceae;Moorea
Hypolith2_3	86.37	3.07	0.00	Bacteria;Cyanobacteria;unclassified;unclassified;unclassified;
Hypolith2_4	96.04	1.33	0.00	Bacteria;Cyanobacteria;Chroococcidiopsidales;Chroococcidiopsidaceae;Chroococcidiopsis
Hypolith2_5	55.71	1.87	12.50	Bacteria;Actinobacteria;Rubrobacteria;Rubrobacterales;Rubrobacter;
Hypolith2_6	46.84	0.61	0.00	Bacteria;Cyanobacteria;Oscillatoriophycideae;Chroococcales;Gloeocapsa;
Hypolith2_7	34.12	0.22	0.00	Bacteria;Cyanobacteria;unclassified;unclassified;unclassified;
Hypolith2_8	17.51	0.00	0.00	No rpS3
Hypolith2_9	13.91	0.00	0.00	Bacteria;Cyanobacteria;unclassified;unclassified;unclassified;

Table S3: All population genomes binned and their checkM statistics and predicted taxonomy based on rpS3.

	CheckM Statistics			51 Bacterial Single Copy Genes		Genome Statistics						Taxonomy (based on Ribosomal Protein S3)
MAG	Completeness (%)	Contamination (%)	Strain heterogeneity (%)	Completeness (%)	Contamination (%)	iRep	Size (Mbp)	GC content (%)	Coverage	Scaffolds	N50	
Hypolith1_1	99.26	2.67	7.69	98	3.9	1.452	6.0	44.23	26.97	292	86602	Bacteria;Cyanobacteria;Chroococcidiopsidales;Chroococcidiopsidaceae;Chroococcidiopsis
Hypolith1_2	93.48	3.37	13.04	98	1.9	1.750	5.4	48.65	30.19	691	15589	Bacteria;Cyanobacteria;unclassified;unclassified;unclassified;
Hypolith1_3	82.11	2.30	23.08	98	1.9	1.654	4.8	46.40	11.68	380	15245	Bacteria;Cyanobacteria;Chroococcidiopsidales;Chroococcidiopsidaceae;Chroococcidiopsis
Hypolith1_4	82.97	0.79	0.00	92	5.9	2.034	4.3	67.89	5.58	281	26507	Bacteria;Actinobacteria;Rubrobacteria;Rubrobacterales;Rubrobacter;
Hypolith2_1	99.41	3.84	9.09	98	5.8	1.716	7.8	44.56	14.65	708	64242	Bacteria;Cyanobacteria;Chroococcidiopsidales;Chroococcidiopsidaceae;Chroococcidiopsis
Hypolith2_2	90.74	0.71	0.00	96	3.9	1.681	3.6	50.92	5.91	227	35294	Bacteria;Cyanobacteria;Oscillatoriophycideae;Oscillatoriales;Oscillatoriaceae;Moorea
Hypolith2_3	86.37	3.07	0.00	96	0.0	1.790	6.6	47.98	5.74	740	19297	Bacteria;Cyanobacteria;unclassified;unclassified;unclassified;
Hypolith2_4	96.04	1.33	0.00	98	3.9	1.858	5.2	46.02	37.68	982	8550	Bacteria;Cyanobacteria;Chroococcidiopsidales;Chroococcidiopsidaceae;Chroococcidiopsis
